# TORC2—a new player in genome stability

**DOI:** 10.15252/emmm.201403959

**Published:** 2014-07-03

**Authors:** Ronit Weisman, Adiel Cohen, Susan M Gasser

**Affiliations:** 1 Department of Natural and Life Sciences, The Open University of IsraelRaanana, Israel; 2 Friedrich Miescher Institute for Biomedical ResearchBasel, Switzerland

**Keywords:** cancer therapies, DNA damage, mTOR, TORC1, TORC2

## Abstract

The inhibition of the central growth regulatory kinase TOR, which participates in two complexes, TORC1 and TORC2, has been a focus of metabolic and cancer studies for many years. Most studies have dealt with TORC1, the canonical target of rapamycin, and the role of this complex in autophagy, protein synthesis, and cell growth control. Recent work on TORC2 in budding and fission yeast species points to a conserved role of this lesser-known TOR complex in the survival of DNA damage. In budding yeast, TORC2 controls lipid biosynthesis and actin cytoskeleton through downstream AGC kinases, which are now, surprisingly, implicated in the survival of oxidative DNA damage. Preliminary data from mTORC2 modulation in cancer cells suggest that an extension to human chemotherapy is worth exploring.

## Introduction

TOR (target of rapamycin) is an atypical serine/threonine protein kinase that belongs to the family of phosphatidyl inositol-3 kinase related kinases (PI3K-like kinases or PIKKs). TOR proteins are best known for their roles in the nutrient-dependent signaling pathways underlying cell growth, proliferation, and survival. TOR was first identified in the budding yeast *Saccharomyces cerevisiae* as the molecular target of the immunosuppressive and anti-cancer drug rapamycin (Heitman *et al*, 1991). Later, TOR genes were isolated in all eukaryotes investigated. A breakthrough in our understanding of the TOR-dependent signaling pathway coincided with the identification of two distinct TOR-containing complexes, termed TOR complex 1 and TOR complex 2, or TORC1 and TORC2 (Loewith *et al*, 2002; Wedaman *et al*, 2003). Both complexes are conserved from yeast to man (reviewed in Wullschleger *et al*, 2006; Loewith & Hall, 2011).

TORC1 primarily regulates growth and is involved in the modulation of protein synthesis, ribosome biogenesis, and autophagy. Accordingly, disruption of TORC1 in many eukaryotes including *S. cerevisiae*, *Schizosaccharomyces pombe*, nematodes, flies, or mammals results in cellular phenotypes that resemble starvation (for examples, see Barbet *et al*, 1996; Noda & Ohsumi, 1998; Zhang *et al*, 2000; Meissner *et al*, 2004; Alvarez & Moreno, 2006; Matsuo *et al*, 2007; Weisman *et al*, 2007).

The cellular functions of TORC2 are less well understood. This is partly due to the fact that—in contrast to TORC1—there are no specific inhibitors of TORC2 (Sparks & Guertin, 2010). Rapamycin and a family of derivatives that function similarly (“rapalogs”) inhibit only TORC1 and have allowed in-depth analysis of its function in tissue culture cells and multicellular organisms. Rapalogs form a complex with the FK506 binding protein-12 (FKBP-12), which is bound by mTOR, and blocks mTORC1 activity. This in turn inhibits cell cycle progression, cell survival, and angiogenesis. Rapamycin derivatives have been successfully used to treat neurological and metabolic disorders, as well as some cancers, such as renal cell carcinoma, subependymal giant cell astrocytoma associated with tuberous sclerosis, pancreatic and neuroendocrine tumors, and ER^+^ breast cancer (Dienstmann *et al*, 2014; Porta *et al*, 2014). This success uncovered significant crosstalk between the TORC1 complex and the PI3K and Akt/PKB signaling pathways (Dazert & Hall, 2011; Laplante & Sabatini, 2012). Indeed, other pan-PIKK inhibitors that target collectively the related catalytic domains of mTOR and PI3K (examples being Novartis' NVP-BEZ235, and GDC-0980 from Roche/Genentech) have also had clinical success. These and related pan-PIKK/mTOR inhibitors are now being tested on advanced solid tumors, breast cancer, leukemias, and pancreatic and neuroendocrine tumors. Finally, there are also ATP-competitive inhibitors that inhibit only the two mTOR-containing complexes, mTORC1, and mTORC2, although have yet to yield positive clinical results (Dienstmann *et al*, 2014).

In contrast to the starvation-like phenotypes observed upon disruption or inhibition of TORC1, the loss of TORC2 generates diverse effects that often show species- or cell-type specificity. Remarkably, however, two recent reports showed that TORC2 plays a role in the maintenance of genome stability in face of oxidative or replicative stress, in both the fission yeast, *S. pombe,* and the budding yeast, *S. cerevisiae* (Schonbrun *et al*, 2013; Shimada *et al*, 2013). The similarity of these findings in such distantly related yeast reinforces the concept that TORC2 controls a network that might either directly or indirectly safeguard the genome from DNA damage. Here, we briefly review the cellular functions attributed to TORC2 in yeast and discuss the possibility that TORC2 helps ensure genomic stability in higher eukaryotes as well.

## Conserved aspects of TORC1 and TORC2 complexes

**Figure 1:**
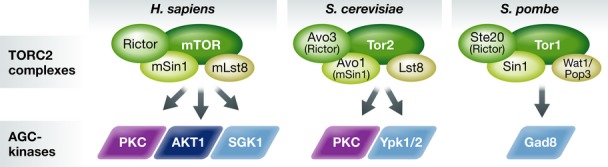
TORC2 complex signaling to AGC kinases Shown is a summary of TORC2 in human, *S. cerevisiae* and *S. pombe*. The human proteins Rictor and Sin1 are TORC2-specific subunits conserved in both yeast species, being known as Avo3 (Rictor) and Avo1 (Sin1) in *S. cerevisiae* (Loewith *et al*, 2002) or Ste20 (Rictor) or Sin1 in *S. pombe* (Hayashi *et al*, 2007; Matsuo *et al*, 2007). The target kinases of TORC2 are AKT1 (Sarbassov *et al*, 2005), SGK1 (Garcia-Martinez & Alessi, 2008), PKC-α (Facchinetti *et al*, 2008; Ikenoue *et al*, 2008), and PKC-ζ (Li & Gao, 2014) in higher eukaryotes, while they are Ypk1 and Ypk2 (Kamada *et al*, 2005), and the PKC ortholog Pkc1 (Facchinetti *et al*, 2008) in budding yeast. Fission yeast has only one identified AGC-kinase, Gad8 (Matsuo *et al*, 2003).

Mammalian cells contain a single TOR gene (mTOR) that encodes the catalytic subunit of either TORC1 or TORC2, while in *S. cerevisiae*, there are two TOR genes, *TOR1* and *TOR2*. The budding yeast Tor1 is found exclusively in the TORC1 complex, while Tor2 can serve as the catalytic subunit of either complex (Loewith *et al*, 2002; Loewith & Hall, 2011). Like budding yeast, the distantly related fission yeast also carries two TOR genes, *tor1*^*+*^ and *tor2*^*+*^. In *S. pombe,* Tor1 is the catalytic subunit of TORC2, while Tor2 is the catalytic subunit of TORC1, as the genes were named in order of discovery and not based on their function (Hayashi *et al*, 2007; Matsuo *et al*, 2007; Ikai *et al*, 2011). In all species, the two complexes are distinguished by conserved complex-specific subunits. Notably, the human subunits Rictor and Sin1 are TORC2-specific and are conserved in both yeast species, albeit with different names (Fig 1).

In all species studied to date, TOR complexes appear to work through a common mechanism: the phosphorylation of the protein kinase A/protein kinase G/protein kinase C (AGC) subfamily (Weisman, 2010). TOR kinase activates AGC kinases by phosphorylating their hydrophobic and turn motifs (Jacinto & Lorberg, 2008). As shown in Fig 1, the target kinases are AKT1, SGK1, PKC-α, and PKC-ζ in higher eukaryotes, Ypk1 and Ypk2 and the PKC ortholog Pkc1 in budding yeast, and Gad8 in *S. pombe*. The budding yeast TORC2 complex regulates cell wall integrity, actin polarity, endocytosis, membrane growth, and sphingolipid biosynthesis through Ypk1/2 (reviewed in Jacinto & Lorberg, 2008), while in fission yeast, Gad8 is thought to mediate many, if not most, of TORC2 functions (Fig 1).

## Comparing phenotypes of budding and fission yeast TORC2 mutants

On a phenotypic level, the cellular activities of TORC2 complexes in fission and budding yeasts appear substantially different. It is unclear whether this reflects a true divergence in function or an incomplete understanding of the functions controlled by TORC2. Budding yeast TORC2 is essential for viability, while *Sp*TORC2 is essential only under stress conditions, such as nutrient starvation. The essential cellular functions attributed to *Sc*TORC2 are the regulation of actin polarization and sphingolipid biosynthesis (Schmidt *et al*, 1996; deHart *et al*, 2002; Aronova *et al*, 2008), reviewed in Loewith and Hall (2011). Because polarized actin filaments are needed for the transport of proteins and lipids to the growing daughter cell, actin regulation by the TORC2-Ypk1/2 pathway is crucial for growth in budding yeast. Sphingolipids, which have both structural and signaling functions, are core components of all lipid bilayers and are necessary for bud growth. Not only does *Sc*TORC2 control sphingolipid biosynthesis, but the lipids themselves feedback to regulate *Sc*TORC2 activity (Aronova *et al*, 2008). Furthermore, this complex is activated by plasma membrane stress, which stems from cell surface expansion during growth, or from chemical or mechanical stress on the plasma membrane (Berchtold *et al*, 2012). Finally, one study suggested that contacts between *Sc*TORC2 and ribosomes activate the kinase, a mechanism possibly conserved in human cells (Oh *et al*, 2010; Zinzalla *et al*, 2011).

In *S. pombe,* disruption of *Sp*TORC2 is non-lethal but results in a delayed entrance into mitosis and generates slightly elongated cells (Weisman & Choder, 2001; Ikeda *et al*, 2008; Ikai *et al*, 2011). The fission yeast TORC2 function is essential to execute the two main responses to starvation: sexual development and entrance into stationary phase (Kawai *et al*, 2001; Weisman & Choder, 2001) and is required for both amino acid uptake (Weisman *et al*, 2007) and growth on low glucose (Ikai *et al*, 2011). Loss of *Sp*TORC2 activity rendered cells remarkably sensitive to a variety of environmental insults, including low or high temperature, osmotic, and/or oxidative stress. However, unlike the growth-inhibiting actin polarization defect observed in budding yeast, the *S. pombe* TORC2-Gad8 pathway currently appears to be only loosely connected with actin organization. Nonetheless, an abnormal distribution of actin cortical dots and excess actin polymerization at the cell equator do occur in *S. pombe* cells lacking functional TORC2 (Ikai *et al*, 2011). It may be that the differences in cell growth and actin phenotypes associated with loss of TORC2 in the two yeasts stem from their fundamentally different modes of cell growth and division. *S. cerevisiae* has unidirectional bud emergence, and its growth is strictly controlled by polarized actin filaments, while *S. pombe* growth occurs first at both ends and then shifts to a central cleavage furrow, where it promotes cell membrane and cell wall synthesis (Loewith, 2013).

Transcriptional profiles of *S. pombe* cells lacking the catalytic subunit of TORC2 resemble those of cells lacking histone de-acetylases or chromatin remodeling subunits (Schonbrun *et al*, 2009). Interestingly, disruption of *gad8*^+^ resulted in hypersensitivity to DNA-damaging agents, elongation of telomeres, and loss of gene silencing at the mating-type locus for unknown reasons (Schonbrun *et al*, 2009). Closer examination suggested that the complex was specifically required for cell survival under chronic DNA replication stress (Schonbrun *et al*, 2013).

Although the budding yeast TORC2 had never been directly implicated in the DNA damage response, a chemicogenetic screen carried out in budding yeast identified a novel imidazoquinoline NVP-BHS345 that targeted both *Sc*TORC1 and *Sc*TORC2. NVP-BHS345 conferred a synergistic lethality on cells exposed to oxidation and break-inducing agents like Zeocin or ionizing radiation (Shimada *et al*, 2013). The cells deficient for *Sc*TORC2 showed no signs of DNA damage in the absence of exogenous agents, yet the combination of base-oxidizing damage with loss of either Tor2 or Ypk1/Ypk2 activity generated a genomewide fragmentation called yeast chromosome shattering (YCS), mimicking the effects of NVP-BHS345 and Zeocin (Shimada *et al*, 2013). To a lesser degree, the chemical inhibitor also enhanced sensitivity to replicative stress in strains lacking the RecQ helicase Sgs1, although these conditions did not lead to chromosome fragmentation (Shimada *et al*, 2013).

Response to DNA damage is crucial for cell survival, given that DNA is continuously assaulted by free radicals and damage arising from replication stress. Although many members of the PIKK family, which includes TOR kinases, play pivotal roles in DNA damage response (Carr, 2002; Friedel *et al*, 2009), neither ataxia telangiectasia mutated (ATM) nor ATR (ATM, *Sp*Rad3-related) kinases were responsible for the damage sensitivity observed in TORC2-deficient *S. pombe* or *S. cerevisiae* cells (Schonbrun *et al*, 2013; Shimada *et al*, 2013). Specifically, the ATR homologues (*Sc*Mec1 or *Sp*Rad3) and the effector kinases (*Sc*Rad53 or *Sp*Chk1) were fully functional in face of DNA damage, despite loss of TORC2 activity. Rad53 kinase was even hyperactivated by DNA damage upon TORC2 inhibition (Shimada *et al*, 2013). Nonetheless, disruption of the catalytic subunit of TORC2 or the downstream kinase, Gad8 in *S. pombe* cells, rendered cells sensitive to replication stress induced by hydroxyurea (HU), methyl-methane sulfonate (MMS), or camptothecin (CPT) (Schonbrun *et al*, 2013). Indeed, perturbation of *Sp*TORC2-Gad8 signaling led to cell cycle arrest and elongation in response to DNA damage, consistent with activation of the DNA damage checkpoint response (Schonbrun *et al*, 2013).

The chemical identified in the budding yeast screen, NVP-BHS345, is not a general PIKK family inhibitor in yeast, but selectively inhibits *S. cerevisiae* TORC1 and TORC2. In human cells, on the other hand, the same compound is a broad spectrum inhibitor of the mammalian PIKK family (Shimada *et al*, 2013). The striking fragmentation of yeast chromosomes that occurs when inhibition of *Sc*TORC2 is combined with low-level doses of Zeocin occurs rapidly (< 1 h) and without passage through the cell cycle. Treatment with rapamycin, which inhibits exclusively *Sc*TORC1, did not have similar effects (Shimada *et al*, 2013). Genetic analysis showed that YCS arises specifically from the combination of *Sc*TORC2 inhibition and low levels of oxidative damage. Indeed, a hyperactivating mutation in *YPK2* rendered the cells resistant to the damage provoked by NVP-BHS345 and Zeocin. Collectively, chemicogenetic evidence proved that *Sc*TORC2-dependent activation of Ypk1/Ypk2 participates in Zeocin-induced DNA damage survival (Shimada *et al*, 2013).

Its mechanism might entail inhibition of a repair pathway, as the essential homologues recombination (HR)-protein Rad52 accumulated in foci during the combined treatment. Perturbation of *Sp*TORC2-Gad8 signaling also generated an accumulation of Rad52-containing repair foci. It remained unclear which repair pathway was impaired, although both HR and non-homologous end joining were ruled out (Shimada *et al*, 2013). Consistently, *Sp*TORC2-Gad8 mutants were synthetic lethal with loss of either *S. pombe* Rad52 or Rad51, possibly indicating that *Sp*TORC2-Gad8 mutants incur an enhanced level of DNA damage that renders them dependent on HR. In further support of this, negative synthetic interactions were scored between *Sp*TORC2-Gad8 mutants and deletions of DNA repair or replication fork re-start genes, such as *mus81*, *mms1,* or *mms22*, even in the absence of damage-inducing agents (Schonbrun *et al*, 2013).

Although the fission yeast genetics suggested hypersensitivity to replication stress, the Zeocin-induced YCS in budding yeast did not require entry into S phase. Moreover, a similar sensitivity to low levels of oxidative damage was observed in budding yeast in the presence of the actin polymerization inhibitor, latrunculin A (LatA) or jasplakinolide, a cyclic peptide that stabilizes F-actin, in combination with low levels of Zeocin (Shimada *et al*, 2013). This suggested that either an actin-associated activity or actin filament turnover itself, which is indeed under TORC2-YPk1/2 control, impairs genome integrity under conditions of increased oxidative stress.

In the cytoplasm, actin forms a network of filaments that mediates macromolecular transport and maintains cell morphology. In the cell nucleus, monomeric globular (G) actin has been implicated in functions such as chromatin remodeling and transcription (Belin & Mullins, 2013), but it is unclear what role is played by filamentous (F) actin. There is an active system of actin import and export to and from the nucleus (Vartiainen, 2008), suggesting that nuclear actin concentration is under careful control. Nonetheless, it remains to be determined whether impaired G- to F-actin turnover impacts DNA repair, or otherwise protects the genome, and if that role is lost upon *Sc*TORC2 inhibition.

Interestingly, in *S. pombe*, the loss of *Sp*TORC2-Gad8 led to an increased level of Rad52 foci, yet did not activate the DNA damage checkpoint under normal growth conditions. This characteristic is shared with the *brc1* mutant, which eliminates Brc1, a protein bearing six BRCT domains (Bass *et al*, 2012). This may indicate that the damage incurred by loss of TORC2 is not recognized by the DNA damage checkpoint or else is too low to activate the response. On the other hand, upon removal of DNA-damaging agents, the dephosphorylation of *Sp*Chk1 (termination of the checkpoint signal) and activation of *Sp*Cdc2, the central cyclin-dependent kinase, were delayed (Schonbrun *et al*, 2009, 2013). Further study is required to clarify whether this reflects an accumulation of unrepaired lesions or misregulation of a phosphatase required for *Sp*Chk1 dephosphorylation in the absence of *Sp*TORC2.

## Implications for human disease and therapies

As mentioned above, there is only one Tor kinase subunit in mammalian cells, mTOR, serving in both mTORC1 and mTORC2. It is therefore not surprising that there are no active-site inhibitors that distinguish the complexes, as strictly speaking, the active site is identical in both. Rapamycin and rapalogs do allow specific inhibition of mTORC1 signaling, while specific inhibition of mTORC2 requires shRNA of Rictor, an complex-specific co-regulator.

As mentioned above, a number of PIKK family kinase inhibitors inhibit mTOR along with PI3K and, in some cases, with other related PIKK family members (DNA-PK, ATR and ATM). These and the rapamycin derivative Affinitor® are well validated for combined chemotherapeutic protocols for solid tumors (Beuvink *et al*, 2005; Sparks & Guertin, 2010; Grunt & Mariani, 2013; Tentori *et al*, 2013). Nonetheless, rapalogs do not have broad anti-tumor activity. This may reflect the fact that they only inhibit certain cellular activities of mTORC1 (Shor *et al*, 2009) and can lead to activation of PI3 kinase as a consequence of inhibiting an important negative feedback loop (Wan *et al*, 2007).

**Figure 2:**
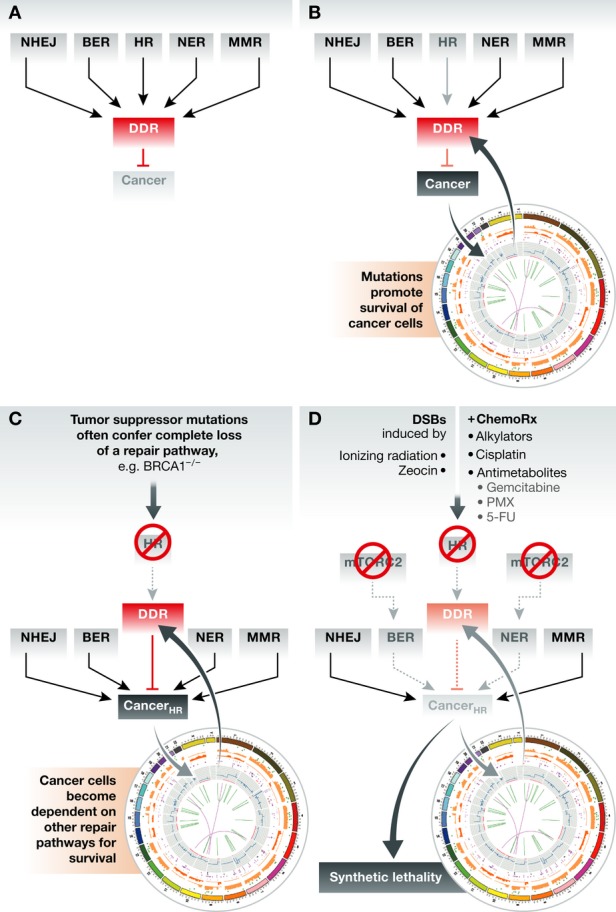
Combinational therapies that impair multiple repair pathways can provoke synergistic lethality in “damage-addicted” cancer cells (A) Multiple repair pathways including non-homologous end joining (NHEJ), base-excision repair (BER), homologous recombination (HR), nucleotide excision repair (NER), and mismatch repair (MMR) protect the cellular genome from damage through conserved pathways that collectively activate the DNA damage response (DDR). (B) In precancerous lesions, overwhelming damage or weakened repair capacity (for example. loss of HR) can lead to selection for mutations that promote survival. Transformed cells often exhibit constitutively activated DDR and enhanced genomic instability. (C) Loss of a repair pathway (through inherited or incurred mutation, e.g. HR) can render cancer cells “addicted” or dependent on the other repair pathways (Cancer_HR_). (D) Combined therapies that are effective against tumors bearing mutant repair pathways often inhibit a second repair pathway. This could be by targeting a general repair factor (e.g. PARP) or—as suggested here—the mTORC2 complex. The resulting accumulation of irreparable damage leads to cell death (synthetic lethality). DNA-damaging agents could enhance the synthetic lethality. Illustration partially adapted (with permission) from Forbes *et al* (2011).

The existence of mechanisms that downregulate mTORC1 upon DNA damage links mTORC1 to the DNA damage response in man (Zoncu *et al*, 2011; Hasty *et al*, 2013). Intriguingly, a recent study revealed physical interaction between the tandem BRCT domain of BRCA1 and Rictor, the mTORC2-specific subunit. BRCA1 is a protein with pleiotrophic effects on genome stability and is a major breast and ovarian susceptibility gene. A set of experiments suggested that BRCA1 binding to Rictor inhibits mTORC2-dependent phosphorylation of Akt-Ser473 (Woods *et al*, 2012). Whether the interactions between BRCA1 and Rictor affect DNA damage tolerance or genome integrity is unknown, although Akt, which is a downstream target of mTORC2 as well as DNA-PK, is linked to the survival of damage (Bozulic *et al*, 2008; Surucu *et al*, 2008). The notion arising from budding yeast, based on the fact that there are few effects of *Sc*TORC2 inhibition alone, is that mTORC2 inhibition might sensitize mammalian cells to radiation or other oxidizing damage to DNA, thus improving standard DNA-damaging chemotherapies (Fig 2). Other more specific DNA-damaging agents, such as *cis*-platin, could also be combined with mTORC2 inhibition. Finally, there may be tumors deficient in specific repair pathways, for which inhibition of mTORC2 would lead to cell death (Fig 2).

The potential benefit of mTORC2 inhibition is suggested by studies that genetically manipulated the levels of Rictor. Deletion of Rictor in mouse models for prostate cancer, or a reduction of Rictor in cancer cell lines, was shown to inhibit tumor development and cell proliferation (Hietakangas & Cohen, 2007; Masri *et al*, 2007; Guertin *et al*, 2009). mTORC2 was also found to be activated in glioblastoma, where it promoted survival and resistance to chemotherapy in a NF-κB-dependent manner (Tanaka *et al*, 2011). To date, no analysis of genome stability under these conditions has been reported.

Second generation mTOR inhibitors that act as ATP-competitive inhibitors of both mTORC1 and mTORC2, or dual specificity inhibitors that target PI3K as well, indirectly suggest a role for mTORC2 in cancer development, possibly by rendering cancer cells more sensitive to replication stress (Yu *et al*, 2009; Falcon *et al*, 2011; Guo *et al*, 2014). In mouse models for T-cell leukemia (Molt-Luc2), treatment with pp242, an mTOR ATP-competitive inhibitor, enhanced DNA damage-induced apoptosis and delayed cancer development (Guo *et al*, 2014). Importantly, treatment of Molt-Luc2 cells with pp242, but not with rapamycin, led to downregulation of the expression of FANCD2, a component of the Fanconi anemia DNA repair complex, which responds to replication fork-associated damage. Consistently, treatment of Molt-Luc2 cells with pp242 (but again, not with rapamycin) sensitized cells to the inhibition of DNA synthesis by cytosine arabinoside (AraC), consistent with a perturbation of recovery from replication stress (Guo *et al*, 2014).

The benefits of simultaneous use of general mTOR inhibitors and DNA-damaging agents to enhance DNA damage-induced apoptosis are well documented (Beuvink *et al*, 2005; Grunt & Mariani, 2013; Tentori *et al*, 2013), reviewed by Hasty *et al* (2013) and Zoncu *et al* (2011). However, it remains to be explored whether the selective inhibition of mTORC2 can improve combined therapy, by sensitizing cancer cells to DNA-damaging agents. Based on the yeast results discussed here, one might expect that targeting mTORC2 will trigger a more selective and synergistic cell death, than pan-mTOR or pan-PIKK inhibitors. The need for more efficient combination therapies against metastatic cancer suggests that this pathway should, indeed, be tested.

**Bridge the gap**

**The gap**

The mTOR kinase subunit is found in two distinct cytoplasmic complexes in mammals, TORC1, and TORC2. It has been difficult to study the second TOR complex in mammals due to the absence of complex-specific inhibitors. Studies in budding and fission yeast have now independently identified a role for the TORC2 complex in the survival of DNA damage. In fission yeast, survival of S phase-specific damage was compromised in strains lacking a functional TORC2 complex. In budding yeast, strong synthetic lethality with oxidizing damage from ionizing radiation or Zeocin was identified in strains treated with a TORC2 inhibitor. To date, there has been no link uniting these diverse results with the potential of using TORC2 as a chemotherapeutic target.

**The bridge**

Here, we explore the evidence arguing that the TORC2 signaling cascade may contribute to resistance or survival in the face of DNA damage in cancer cells, as it does in yeast. It is noteworthy that existing mTOR inhibitor studies are inconclusive with respect to TORC2 as a drug target, because the chemotherapeutic agents used to date are either selective for TORC1 (rapamycin-like inhibitors) or else inhibit not only TORC1 and TORC2, but also PI3 kinases. Given the striking conservation of DNA damage survival phenotypes in budding and fission yeasts, we argue that inhibition of TORC2 may be a novel and fruitful pathway for synthetic chemotherapy of repair-compromised cancers.

## For more information

**List of current clinical anti-cancer trials that include pan-mTOR or pan-PIKK inhibitors**

Roswell Park Cancer Institute: https://www.roswellpark.org/clinical-trials/list

National cancer Institute at the National Institutes for Health: http://www.cancer.gov/clinicaltrials

**List of current clinical trials related to TSC (Tuberous Sclerosis Alliance)**

http://www.tsalliance.org/pages.aspx?content=370

**SGD—*****S. cerevisiae*** **genome database**

http://www.yeastgenome.org/

**PombeBase—*****S. pombe*** **genome database**

http://www.pombase.org/

## Abbreviations

Actin cytoskeleton: Filaments made from a chain of globular actin molecules that reside in the cytoplasm of eukaryotic cells and give the cell its shape and capacity for directed movement.
AGC kinases: A subfamily of serine/threonine protein kinases, including the A, G, and C families of kinases (PKA, PKG and PKC). Other well known members of this family of proteins are Akt, S6K, RSK, and PDK1. A common and highly conserved feature of TOR-mediated signaling pathways is the phsophorylation and activation of specific subset of AGC kinases by TORC1 or TORC2.
AKT kinase: AKT, also known as protein kinase B (PKB), is a member of the AGC kinases that plays a key role in protein synthesis, glucose metabolism, cell proliferation, and survival. AKT can be phosphorylated and activated by mTORC2.
Combination therapy: The use of more than a single therapy or medication to treat a disease, generally cancer. Benefits of combination therapy include reduced rates of drug resistance or toxicity.
DNA damage response (DDR): DNA damage response entails activation of cell cycle checkpoints, DNA repair, and damage tolerance pathways. Failure of DDR causes genome instability that can lead to human syndromes and aging-associated diseases, in particular cancer.
DNA replication stress: Inefficient DNA replication that causes DNA replication forks to progress slowly or stall. DNA replication stress can result from drugs such as hydroxyurea, which depletes the nucleotides pools of DNA intercalating or modifying drugs.
Genome instability: Increased frequency of mutations, chromosomal rearrangements, or aneuploidy. Genome instability is often associated with cancer and can be indicative of a poor prognosis for some types of cancer.
Homologous recombination (HR): A major pathway for DNA damage repair that requires the exchange of DNA sequences between identical and similar (i.e. homologous) molecules of DNA.
Rapamycin: A lipophilic macrolide produced by the soil bacterium *Steptomyces hygroscopicous*. Rapamycin can inhibit cell proliferation of a variety of cells types, an effect that results in anti-fungal, immunosuppressive, or anti-cancer activities.
Rapalogs: Rapamycin and its derivatives, which act through a common pathway to inhibit mTORC1.
Synthetic lethality: Cell death resulting from the combination of two or more mutations that are not lethal when present as single mutations
